# Effect of segmental versus marginal mandibular resection on local and lymph node recurrences in oral squamous cell carcinoma: is tumorous bone infiltration or location and resulting soft tissue recurrences a long-term problem?

**DOI:** 10.1007/s00432-023-04963-0

**Published:** 2023-06-21

**Authors:** Lucas M. Ritschl, Minli Niu, Valeriya Sackerer, Carolina Claßen, Herbert Stimmer, Andreas M. Fichter, Klaus-Dietrich Wolff, Florian D. Grill

**Affiliations:** 1 Department of Oral and Maxillofacial Surgery, School of Medicine, Technical University of Munich, Klinikum Rechts der Isar, Ismaninger Str. 22, 81675 Munich, Germany; 2 Department of Diagnostic and Interventional Radiology, School of Medicine, Technical University of Munich, Munich, Germany; 3 Department of Oral and Maxillofacial Surgery, School of Medicine, University of Saarland, Homburg, Saar, Germany

**Keywords:** Head and neck cancer, Bone infiltration, Mandibular resection, Local recurrence, Lymph node recurrence

## Abstract

**Purpose:**

Oral squamous cell carcinomas (OSCCs) adjacent to the mandible or with clinically suspected bone infiltration are surgically treated either with marginal or segmental resections. This retrospective study compared both resections regarding local recurrence and lymph node recurrence or secondary lymph node metastases.

**Methods:**

All consecutive primary OSCC cases between January 2007 and December 2015 that underwent mandibular marginal or segmental resection were included. Rates of local and lymph node recurrences or secondary metastases and possible risk factors such as tumor localization according to Urken’s classification were recorded.

**Results:**

In total, 180 patients with 85 marginal (group I) and 95 segmental (group II) mandibular resections were analyzed. The local recurrence rates were comparable between the groups (28.2% vs. 27.4%; *p* = 0.897). Lymph node recurrences or secondary metastases were higher in group I (9.4% (*n* = 8) vs. 6.2% (*n* = 6); *p* = 0.001). Tumor localization appears to affect the outcomes. Significantly fewer local and lymph node recurrences/metastases were found for Urken’s classification SB and S calculated by two-proportion z-test (*p* = 0.014 and 0.056, respectively). Local recurrences mostly emerged from soft tissues, which should be resected more radically than the bones.

**Conclusion:**

While bone infiltration appears technically well controllable from an oncologic point of view, local recurrences and lymph node recurrences/metastases remain an issue. Regular clinical aftercare with imaging is crucial to detect recurrences.

## Introduction

Oral squamous cell carcinomas (OSCC) can infiltrate the mandible, and this likeliness for invasion is influenced among others by time (e.g., diagnosis delay), tumor biology, and adjacent localization measuring < 1 cm to the mandible (Pandey et al. 2007; Eichberger et al. 2022). Furthermore, tumorous bone involvement can range from limited cortical erosion to cancellous bone involvement with secondary pathologic fractures. However, the extent of resection and its influence on local recurrence and long-term survival are still being discussed, which results in heterogeneous concepts. These include mandible preservation and periosteal frozen section analysis of OSCC cases that lack cortical erosion in preoperative staging imaging. Marginal mandibular resection is contraindicated in OSCC cases with a radiologically confirmed invasion of the cancellous bone only (Wolff et al. 2012; Abler et al. 2005; Muscatello et al. 2010; Gou et al. 2018). Consequently, most authors agree that a segmental resection is recommended in the case of invasion of the mandibular cancellous bone to achieve a complete, free-margin resection.

Interestingly, the tumor invasion of the mandible appears to have minor prognostic value on the long-term survival of patients with OSCC (Mücke et al. 2011), and the extent of (radical) bone resection (marginal or segmental resection) failed to reveal a significant influence on local recurrence and long-term survival rates (Muñoz Guerra et al. 2003; Gou et al. 2018). However, a positive statistical correlation was reported for intraosseous tumor size and local recurrence rate (Guerra et al. 2003).

Despite these interpretations, local recurrence appears to more likely depend on soft tissue tumorous findings in cases of simultaneous soft tissue and mandibular involvement. Therefore, only segmental resection was recommended in selected cases (O’Brien et al. 2003). This supports the opinion that marginal resection can be as effective as segmental resections in terms of local recurrences (Wolff et al. 2004; Patel et al. 2008; Rao et al. 2012; Brown et al. 2002). However, as the extent of osseous resection has come to a general agreement as stated in the German S3 guideline (Wolff et al. 2012), the role of mucosal recurrences in T4 OSCCs with previous mandibular infiltration was also evaluated critically by Moratin et al. (Moratin et al. 2020). The authors revealed that the localization of tumorous bone invasions is a decisive variable when making decisions for the extent of mandibular resection and recommended that segmental resection is mandatory in anterior tumor localization.

Therefore, this study aimed to evaluate the prognostic value of both mandibular resection types and determine the risk of local recurrence depending on the type of resection and tumor localization. Further, we intended to analyze the correlation between the resection extent and risk of lymph node recurrence or secondary lymph node metastasis given their significant prognostic values (Safi et al. 2018).

## Materials and methods

### Ethical statement

All clinical investigations were conducted according to the principles expressed in the Declaration of Helsinki. This retrospective study was approved by the Institutional Ethics Committee of the Technical University of Munich, Klinikum rechts der Isar (Approval no. 254/22 S-NP).

### Patient population and treatment algorithm

All consecutive primary OSCC cases between January 2007 and December 2015 that underwent mandibular marginal or segmental resection at our department were included in this study. Patients who had recurrent tumors, positive history of radio-/radiochemotherapy in the head and neck area, or previous operations were excluded.

All patients underwent primary surgical treatment after preoperative staging. Based on clinical observation and computed tomography (CT), morphological signs of cortical erosion, or evident bone infiltration, resection procedures included marginal (group I) or segmental (group II) mandibulectomies. Marginal resection was performed if the tumor was attached to the bone surface or in the case of limited cortical erosion. Segmental resection was performed in the case of extensive tumor infiltration with cancellous bone involvement.

### Investigated parameters

Data collection included sex, age, TNM classification, type of neck dissection, reconstruction method, preoperative CT and postoperative pathological findings, surgical duration (min), length of hospital stay (days), and local recurrence rate. According to the guidelines and preoperative decision of the tumor board, neck dissection was conducted in level I–III ipsilateral of the tumor location. In case of intraoperatively detected positive lymph nodes an extended neck dissection was performed in levels IV and V ipsilateral and levels I–III contralateral. In cases with tumors crossing the centerline, neck dissection levels I–III were conducted on both sides. Tumor localizations were categorized corresponding to Urken’s classification describing defects. TNM staging was based on the seventh and eighth editions of the UICC TNM staging system and on postoperative pathological findings. T4a tumors involved both medullary mandibular and soft tissue extension.

Additionally, subgroups were defined as follows:

(A) With regard to radiological bone involvement based on preoperative CT staging imaging: r0, tumor without bone contact; r1, tumor attached to the bone; r2, tumor erosion/infiltration of the bone.
(B) With regard to histologically confirmed bone involvement: h0, no infiltration of the bone; h1, tumor erosion/infiltration of the bone.
(C) With regard to radiological lymph node status based on preoperative CT staging imaging: rlk0, unobtrusive lymph nodes; rlk1, suspicious lymph nodes; rlk2, metastatic lymph nodes.
(D) With regard to histologically confirmed lymph node involvement: plk0, negative lymph node involvement; plk1, positive lymph node involvement.

### Statistical analysis

To calculate the differences between groups I and II with regard to the local recurrence rate, two-proportion z-tests were used. For datasets following a normal distribution, such as time of postoperative local recurrence, surgical duration, and length of hospital stay, unpaired *t* tests were used. The correlation between preexisting risk factors and outcome variables was assessed by logistic regression analysis. The statistical analysis was conducted using IBM SPSS Statistics for Windows, Version 25 (IBM Corp., Armonk, NY, USA), and *p* values < 0.05 were considered statistically significant. Lymph node recurrences and secondary lymph node metastasis were summarized to calculate possible clinically relevant differences caused by an expected low statistical power. G-Power (version 3.1.9.4) was used for power calculation for the analysis of lymph node recurrences/secondary lymph node metastasis.

## Results

### Study population

A total of 180 patients (48 female [27%], 132 male [73%]) with primary OSCC who underwent marginal (group I, *n* = 85) or segmental (group II, *n* = 95) mandibular resection were included in this study. The median age of the patients was 61 (27–82) years; 75.2% of the patients were smokers, 7.1% had type II diabetes, and 61% indicated alcohol abuse. As regards the American Society of Anesthesiologists status, 13.5% were in class I, 67.4% in class II, 19.1% in class III, and 0% in class IV.

The patients were reconstructed according to the reconstructive standard after oncological resection. Here, group I was reconstructed with different microvascular grafts and group II either with alloplastic titanium plate plus microvascular flap or with a microvascular bone flap. Bony mandibular reconstruction with a microvascular bone graft (fibula or iliac crest) was performed freehand, partially adjusted with a resection guide (KLS ReconGuide), or CAD/CAM planed (Weitz et al. 2023).

Specifications of groups I and II with regard to the tumor localization (Urken’s classification), TNM classification, grading, resection status, and adjuvant therapy regimen are presented in Table 1. Logistic regression analysis of T-classification and resection type (marginal vs. segmental) showed statistical significance (regression coefficient, 1.104; *p* < 0.001).

**Table 1:** Specifications of the marginal and segmental resection groups with regard to tumor location, TNM classification, grading, and resection status

	Marginal resection (group I) *n* (%)	Segmental resection (group II) *n* (%)	Total
Type of resection	85 (47.2%)	95 (52.8%)	180
*Tumor localization* (Urken’s classification)			
B (body)	38	50	92
S (symphysis)	25	21	46
SB (symphysis and body)	14	9	23
BSB (body–symphysis–body)	0	3	3
RB (ramus and body)	2	1	3
*TNM classification*			
T1	25	5	30
T2	29	12	41
T3	12	6	18
T4a	18	67	85
T4b	0	1	1
N0	59	49	108
N1	11	5	16
N2a	0	1	1
N2b	8	21	29
N2c	4	12	16
*Grading*			
G1	9	1	10
G2	56	63	119
G3	16	28	44
*Resection status*			
R0	74	78	152
R1	3	9	12
R2	1	0	1
Rx	4	4	8
*Adjuvant therapy*			
No adjuvant therapy	36	7	43
Adjuvant radiotherapy	40	64	104
Adjuvant radiochemotherapy	9	24	33

The distribution of patients with marginal and segmental resection with regard to the results of preoperative CT and postoperative pathological findings are shown in Table 2.

**Table 2:** Distribution of patients with marginal and segmental resections with regard to preoperative CT, postoperative pathological findings, and adjuvant therapies

	Marginal resection (group I) *n*	Segmental resection (group II) *n*	Total
**Preoperative CT**			
*Bone involvement*			
Tumor without bone contact (r0)	24	6	30
Tumor attached to the bone (r1)	38	17	55
Tumor erosion/infiltration of the bone (r2)	10	64	74
*Lymph nodes*			
Unobtrusive lymph nodes (rlk0)	16	10	26
Suspicious lymph nodes (rlk1)	38	38	76
Metastatic lymph nodes (rlk2)	16	35	51
**Postoperative histological findings**			
*Bone involvement*			
No infiltration of the bone (h0)	61	16	77
Tumor erosion of the bone (h1)	2	5	7
Tumor infiltration of the bone (h2)	22	76	98
*Lymph nodes*			
Negative lymph nodes (plk0)	0	1	1
Positive lymph nodes (plk1)	59	47	106

### Surgical duration and hospitalization

**Fig. 1:**
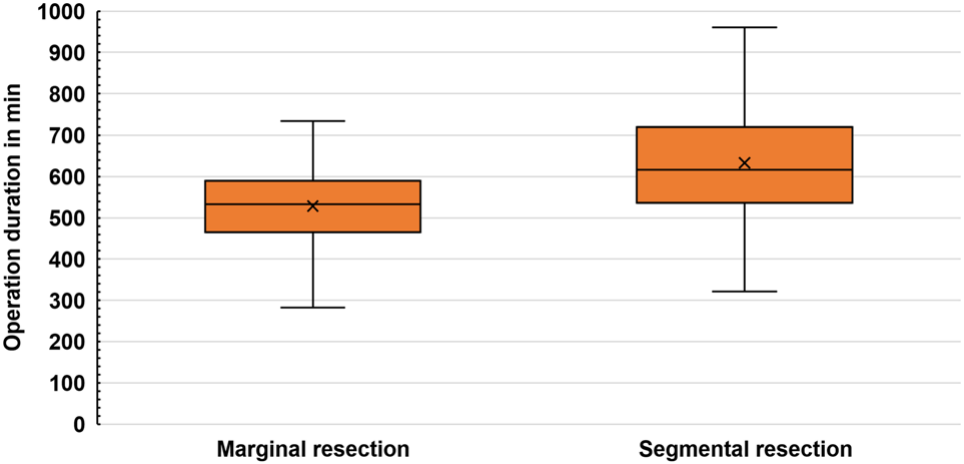
Operation duration of different resection types. Surgical duration (in minutes) in the marginal (I) and segmental (II) resection groups. Values between the two groups were statistically significant (*p* < 0.001)

**Fig. 2:**
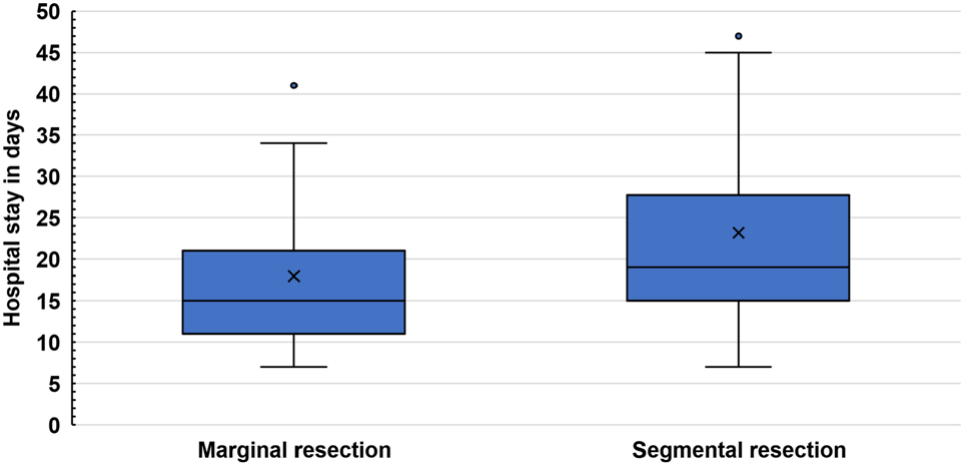
Duration of hospital stay of different resection types. Length of hospital stay (in days) in the marginal (I) and segmental (II) resection groups. Values between the two groups were statistically significant (*p* = 0.023)

The total median operation time of all patients was 573 (282–960) min. The median operation time of group II was significantly higher than that of group I [616.5 (321–960) and 533 (282–735) min, respectively; p < 0.001]. Moreover, group II had a significantly longer hospital stay [19 (7–81) and 15 (7–100) days, respectively; *p* = 0.025] (Figs. 1 and 2).

### Local recurrence rate

The local recurrence rates for groups I and II in total and subgroups with regard to preoperative CT and postoperative pathological findings are presented in Table 3. In total, the local recurrence rate in group I was similar to that of group II (28.2% and 27.4%; *p* = 0.897). Of 52 recurrent cases, 44 showed tumor recurrence arising from soft tissues, whereas only six cases showed bone recurrence alongside soft tissue involvement. Groups I and II comprised three recurrences with bone and soft tissue involvement each. Soft tissue recurrences were detected in 12 cases in group I and in 32 cases in group II. Group II had a higher local recurrence rate than group I (26.7% vs. 22.2%, *p* = 0.733) in subgroup r1 (radiological tumor attachment to the bone). In the same subgroup r1, patients without histologically confirmed pathological bone involvement presented similar tendencies of the local recurrence rate (37.5% vs. 20%, *p* = 0.301), such as patients with histologically positive lymph nodes (33.3% versus 23.1%, *p* = 0.745).

**Table 3:** Local recurrence rate in the marginal and segmental resection groups in total and regarding preoperative computed tomography and postoperative pathological findings

Local recurrence rate	Marginal resection (group I) *n* (%)	Segmental resection (group II) *n* (%)	Two-proportion z-test
In total	24 (28.2%)	26 (27.4%)	0.897
Subgroup with r1	8	4	0.733
Subgroup with r1 and h0	6	3	0.301
Subgroup with r1 and h1	2	2	0.733
Subgroup with r1 and plk1	6	5	0.745
Subgroup with r0	7	1	1.000
Subgroup with r2	4	16	0.321
Subgroup with h0	17	8	0.119
Subgroup with h1	6	19	0.829

Lymph node recurrence rate/secondary lymph node metastasis rate	Marginal resection *n* (%)	Segmental resection *n* (%)	Two-proportion z-test
In total	8 (9.4%)	6 (6.2%)	0.001*
Subgroup with r0	2	0	0.464
Subgroup with r1	3	1	0.166
Subgroup with r2	1	6	0.024*
Subgroup with h0	5	2	*p* < 0.001*
Subgroup with h1	3	5	*p* < 0.001*

Radiological bone involvement: r0, tumor without bone contact; r1, tumor attached to the bone; r2, tumor erosion/infiltration of the bone. Histological bone involvement: h0, no infiltration of the bone; p1, tumor erosion/infiltration of the bone; plk1, positive lymph nodes.

For the calculation of differences between the two cohorts with regard to the local recurrence rate and lymph node recurrence rate, two-proportion z-tests were used. *p values < 0.05 were considered statistically significant.

Patients in the same subgroup with histologically confirmed tumor erosion/infiltration of the bone (h1) showed a higher local recurrence rate following marginal resections (33.3% versus 25%; *p* = 0.733). Contrarily, cases without bone contact in preoperative CT scans (subgroup r0), cancers with radiological diagnosis (subgroup r2), and those with histologically confirmed tumor erosion/bone infiltration (subgroup h1) had a higher local recurrence rate if they underwent marginal resection (29.2% versus 16.7%, *p* = 1.000; 40% versus 25%, *p* = 0.321; 27.3% versus 25%, *p* = 0.892). In contrast, patients in group II without any histologically confirmed bone infiltration (subgroup p0) showed a higher local recurrence rate (53.3% versus 31.5%; *p* = 0.119). The mean postoperative local recurrence time was 22.1 ± 21.3 months in group I and 20.1 ± 24.5 in group II (*p* = 0.766) and occurred 24 times in group I and 26 times in group II.

### Lymph node recurrence/secondary lymph node metastasis

The calculated statistical power with an expected effect size of 0.1 only reached a very low level of power (0.16; G-Power Version 3.1.9.4). Therefore, in the analysis, no difference was found between lymph node recurrence and secondary lymph node metastasis. In total, the rates of lymph node recurrence and secondary lymph node metastasis in group I were significantly higher than those in group II (9.4% [*n* = 8] and 6.2% [*n* = 6], respectively; *p* = 0.001). All six patients in group II and seven out of eight patients in group I received adjuvant radio(chemo)therapy. Four of six patients in group II and three of eight patients in group I had lymph node recurrences, and the other half of the patients had secondary lymph node metastases. In the r0, r1, and h0 subgroups, group I had a higher lymph node recurrence rate/secondary lymph node metastasis rate than group II (8.3% and 0%, *p* = 0.464; 8.3% and 6.3%, *p* = 0.166; 9.3% and 12.5%, *p* < 0.001, respectively). However, subgroups r2 and p1 showed significantly higher rates of lymph node recurrence/secondary lymph node metastasis in group II (10% versus 9.2%, *p* = 0.024; 13.6% versus 6.5%, *p* < 0.001, respectively).

### CT detection of pathological lymph nodes

Preoperative detection of pathological lymph nodes by CT scans showed a sensitivity of 99.2% and a specificity of 100% (Table 4).

**Table 4:** Time of postoperative local recurrence in the group of marginal and segmental resection and sensitivity and specificity of CT detection of pathological lymph nodes

	Marginal resection (group I)	Segmental resection (group II)	*T* test
Mean postoperative local recurrence time (months)	22.1	20.1	0.766
Mean postoperative lymph node recurrence time (months)	10.0	12.6	0.667
	Sensitivity	Specificity	
CT detection of pathological lymph nodes	99.2%	100%	

For datasets following a normal distribution such as time of postoperative local recurrence and lymph node recurrence, unpaired *t* tests were used. *p values < 0.05 were considered statistically significant. CT, computed tomography.

### Predictive factors for local and lymph node recurrence rate or secondary lymph node metastasis rate

Logistic regression was performed to analyze factors potentially predictive of the local recurrence rate, including the resection type, positive/negative lymph nodes, adjuvant therapy, or tumor location. A significant association was found between local recurrences and effectiveness of adjuvant radiotherapy and adjuvant radiochemotherapy (regression coefficient, 0.599, *p* = 0.042; Table 5). Furthermore, tumor location corresponding to Urken’s classification in total and tumor location corresponding to groups S and SB showed a significant correlation with the local recurrence rate (regression coefficients, − 0.738, − 1.099, and − 2.303; *p* = 0.004, 0.002, and 0.002, respectively). None of the patients in the BSB group suffered from local recurrence. Clinically relevant influencing factors such as resection type, positive/negative lymph nodes, or tumor size according to T-classification did not show any statistical significance in the logistic regression (Table 5). Logistic regression was also performed to detect the correlation between the rates of lymph node recurrence or secondary lymph node metastasis and the clinically relevant influencing factors mentioned above. Significances were detected between the rates of lymph node recurrence or secondary lymph node metastasis and tumor location analog to Urken’s classifications B, S, and SB (regression coefficients, − 1.997, − 3.807, and − 2.351; p < 0.001, 0.001, and 0.001, respectively). None of the patients in the BSB group suffered lymph node recurrence. Tumor location in total and other clinically relevant influencing factors did not show statistical significance in the logistic regression (Table 5).

**Table 5:** Logistic regression analysis of the local recurrence rate, lymph node recurrence rate/secondary lymph node metastasis rate, and clinically relevant influencing factors

	Local recurrence		Lymph node recurrence rate/secondary lymph node metastasis rate	
	Regression coefficient	*p* value	Regression coefficient	*p* value
Resection type (marginal or segmental resection)	− 0.096	0.778	− 0.014	0.983
Lymph node involvement (positive or negative)	− 0.322	0.387	− 0.500	0.438
Adjuvant therapy (no therapy, adjuvant radiotherapy, or radiochemotherapy)	0.599	0.042*	0.155	0.753
Tumor location (Urken’s classification B, S, SB, BSB, and RB)	− 0.738	0.004*	− 0.216	0.522
T-classification (T1, T2, T3, T4a, or T4b)	0.031	0.832	0.009	0.970

The correlation of clinically relevant influencing factors and local recurrence rate was assessed by logistic regression. Adjuvant therapies are either radiotherapies or radiochemotherapies

B, body; S, symphysis; SB, symphysis and body; BSB, body–symphysis–body; RB, ramus and body

*p values < 0.05 were considered statistically significant. The correlation of clinically relevant influencing factors and lymph node recurrence rate was assessed by applying a logistic regression. Adjuvant therapies are either radiotherapies or radiochemotherapies. None of the patients in the BSB group have suffered local or lymph node recurrence. *p values < 0.05 were considered statistically significant

The rates of local and lymph node recurrence/secondary lymph node metastasis according to tumor location are presented in Table 6. According to Urken’s classification, the SB group had a significantly lower local recurrence rate than the B group or in all patients (*p* = 0.004 and 0.014, respectively). Furthermore, the B group presented a higher local recurrence rate than the S group and all patients (*p* = 0.062 and 0.813, respectively). The RB group had a slightly higher and the S group a slightly lower local recurrence rate than other groups.

**Table 6:** Local recurrence rate and lymph node recurrence rate/secondary lymph node metastasis rate in different tumor location classes corresponding to Urken’s classification

	Tumor location (Urken’s classification) *n* (%)	Tumor location (Urken’s classification) *n* (%)	Two-proportion *z*-test
Local recurrence rate	B 35 (41.7%)	S 11 (25%)	0.062
	B 35 (41.7%)	SB 2 (9.1%)	0.004*
	B 35 (41.7%)	RB 1 (50%)	0.813
	B 35 (41.7%)	S, SB, BSB, and RB 14 (19.7%)	0.813
	S 11 (25%)	SB 2 (9.1%)	0.126
	S 11 (25%)	RB 1 (50%)	0.431
	S 11 (25%)	B, SB, BSB, and RB 38 (34.5%)	0.251
	SB 2 (9.1%)	RB 1 (50%)	0.094
	SB 2 (9.1%)	B, S, BSB, and RB 47 (35.3%)	0.014*
	RB 1 (50%)	B, S, SB, and BSB 48 (31.4%)	0.574
Lymph node recurrence rate/secondary lymph node metastasis rate	B 11 (12%)	S 1 (2.2%)	0.055
	B 11 (12%)	SB 2 (9.1%)	0.704
	B 11 (12%)	RB 1 (33.3%)	0.273
	B 11 (12%)	S, SB, BSB, and RB 4 (5.4%)	0.143
	S 1 (2.2%)	SB 2 (9.1%)	0.194
	S 1 (2.2%)	RB 1 (33.3%)	0.008*
	S 1 (2.2%)	B, SB, BSB, and RB 14 (11.7%)	0.056
	SB 2 (9.1%)	RB 1 (33.3%)	0.225
	SB 2 (9.1%)	B, S, BSB, and RB 13 (9%)	0.992
	RB 1 (33.3%)	B, S, SB, and BSB 14 (8.6%)	0.139

For the calculation of differences between the two cohorts with regard to the local recurrence rate or secondary lymph node recurrence rate, two-proportion z-tests were used. None of the patients in the BSB group have suffered local or lymph node recurrence

B, body; S, symphysis; SB, symphysis and body; BSB, body–symphysis–body; RB, ramus and body

*p values < 0.05 were considered statistically significant

Group S showed fewer lymph node recurrences/metastases than the overall cohort and the B group (*p* = 0.055 and 0.056, respectively) and significantly fewer lymph node recurrences/metastases than the RB group (*p* = 0.008). The SB group had slightly lower and the RB and B groups slightly higher lymph node recurrence/secondary lymph node metastasis rates than other groups (Table 6).

## Discussion

The tumor size and expected biomechanical stability of the remaining mandible determine the extent of the resection—marginal (I) versus segmental (II). According to our results there was a significant correlation between tumor size (T-classification) and resection type (marginal vs. segmental). Our data showed that segmental resections not only required significantly longer operation time but also led to a longer hospital stay (*p* < 0.001 and 0.025, respectively). Furthermore, segmental resections also bear a significant loss in the quality of life of patients (Dholam et al. 2019; Rogers et al. 2004). Still, with regard to recurrences, few compromises in the case of mandibular infiltrations are possible (Gou et al. 2018; Li et al. 2017).

### Local recurrences

The local recurrence rates of both resection types (I versus II) were similar and comparable with the findings of other studies (Mücke et al. 2011; Wolff et al. 2004; Stoop et al. 2020). Cases with histological confirmation of preoperative radiological tumor proximity to the mandible (r1) were associated with a higher local recurrence rate, which was not statistically significant but remains clinically relevant. However, our results indicated that cortical erosion due to tumor progression had better outcomes than cancellous bone involvement (Li et al. 2017). Further, in cases with limited tumor involvement, marginal resections were reported to be safe with respect to recurrences (Muscatello et al. 2010; Gou et al. 2018; Guerra et al. 2003) in the underlying cortical bone, which serves as a possible protective barrier (Mücke et al. 2011; Li et al. 2017; Ash et al. 2000). In case of cancellous tumor infiltration, segmental resections were recommended regarding the survival rate (Gou et al. 2018). No higher local recurrence rates were detected in cases without histological tumor invasion (h0). If radiologically depicted tumor appears close to the mandible, periosteal fresh-frozen sections may be the answer to deciding whether a segmental resection must be performed (Wolff et al. 2012). This was also emphasized by Brown and Lewis, who summarized in their review that marginal resections can reach a safe resection margin; however, in 15.5% of the patients, bone invasion was noted although not primarily suspected. According to their conclusion, the extent of resection should be based on clinical observation and at least two imaging techniques (Brown et al. 2001). As the assessment of CT scans is impaired by metal artefacts, magnetic resonance imaging is an optimal additional imaging technique alongside CT because it can provide additional important information regarding the extent of cancellous bone involvement (Wolff et al. 2012). Furthermore, PET-CT represents a useful supplement for diagnosing bone invasion besides the imaging modalities mentioned above (Lin et al. 2021). Thus, over- or undertreatment due to imprecise preoperative radiological findings can be increasingly avoided. This aspect can be improved in our workflow, as our retrospective analysis shows; however, only three fresh-frozen sections were taken and two of them were positive for OSCC infiltration. Therefore, a consequent segmental resection, instead of a consecutive marginal resection, should have been decided in these cases.

The local recurrence rate was higher in group I, especially in cases with radiologically diagnosed and histologically confirmed tumor invasion. Even without statistical significance it was clinically plausible (*p* = 0.733). This finding is supported by the results of other authors (Mücke et al. 2011; Rogers et al. 2004; Ash et al. 2000). Interestingly, the local recurrence rate in cases without postoperative histological osseous tumor invasion was greater and close to statistical significance in group II (*p* = 0.072). These, partly contradicting, findings led us back to the fact that most local recurrences in our patients were found in the soft tissues. Segmental resections were mostly planned preoperatively in cases with at least cT2 tumor stage (cortical erosion) or cT4 (bone infiltration), which also have a great extent of soft tissues. Despite the risk of recurrences in higher T-stages, this was not detected to have a statistically significant influence in other investigations (Patel et al. 2008; Stoop et al. 2020). According to our results, the greatest risk of local recurrence was not due to the bone but to soft tissue recurrence. As the evaluation of free bony resection margins appears technically well controllable from an oncologic point of view with the use of intraoperative cytological assessment (Nieberler et al. 2020), local recurrences of soft tissues and lymph node recurrences/metastases remain an issue. Thus, tumor resections should be conducted less invasively in the bone (segmental resections should only be considered in case of medullar bone infiltration) and more radically in soft tissues. Although recurrences mostly emerged from the soft tissue in our study, the potential likelihood of insufficient resection because of CAD/CAM surgery with fewer options to react easily should be further investigated. But none of these studies found a significant correlation between resection method and overall survival rate (Lin et al. 2014; Ma et al. 2022; Camuzard et al. 2017). In our opinion we would rather resect more bone than necessary, because we will achieve a good reconstruction anyway in CAD/CAM cases. In fact, the described risk factors like presence of advanced tumor stage, positive/close surgical margins, or nodal, vascular, or perineural invasion have a greater impact on survival rate than the type of bony tumor resection and reconstruction (Lin et al. 2014; Ma et al. 2022; Camuzard et al. 2017).

### Lymph node recurrences or secondary lymph node metastases

Our results showed that segmental resections resulted in higher rates of lymph node recurrences or secondary lymph node metastases in cases with radiologically diagnosed and histologically confirmed bone infiltration (*p* = 0.024 and p < 0.001, respectively). As extended tumors with higher T-classification have a greater risk of distant or lymph node metastases, this patient population must be closely monitored after surgery. For safe detection of lymph nodes, alongside physical examination, which can be difficult in irradiated necks, objective three-dimensional imaging appears necessary. The calculated specificity of 100% exceeds even the results from ultrasonography (Ash et al. 2000) and supports our suggestion. The significantly higher rates of lymph node recurrences or metastases even in subgroups r0, r1, and h0 can lead to the assumption that occult lymph node metastasis is possibly more likely to occur in large tumors with close bone proximity but without infiltration. Therefore, close cervical monitoring is necessary (Pentenero et al. 2005; Vassiliou et al. 2020). Nevertheless, the emergence of occult lymph node metastasis appears to be not only dependent on tumor size, but also on multiple, genetic, and tumor biological factors (Méndez et al. 2011; Myo et al. 2005).

### Predictive factors for recurrences

In the logistic regression, the calculated statistically significant risk factors turned out to be (a) adjuvant therapy and (b) tumor localization for local recurrences. Concerning lymph node recurrences or metastases, none of the calculated risk factors reached statistical significance. Further, the lower rates of tumor infiltration localization of SB and S according to Urken’s classification (Brown et al. 2016) reached statistical significance in further analyses for local and lymph node recurrences or metastases. Moratin et al. already reported that localization might influence local and lymph node recurrences/metastases. Unlike our findings, which indicate the body and ramus (B and RB) to be an area of risk, Moratin et al. found the anterior mandible (S) to have an increased risk of local recurrence (Moratin et al. 2020). In our study, tumors with greater extension from the symphysis to the body of the jaw (SB) showed significantly fewer local recurrences and tendencies for fewer lymph node recurrences or metastases. This might be due to the greater extent of tumor/soft tissue resection. Furthermore, the anterior region (S and SB) provides better access and overview for tumor resection than the posterior region (B and RB).

### Limitations

The retrospective design of this study has known weaknesses. Even in our head and neck cancer department, the mandible is affected in only a small fraction of patients with tumors. This makes large numbers hardly achievable in a prospective study in a reasonable time frame. Moreover, the eight-year study period might lead to the assumption that any changes to the team of surgeons can influence surgical outcomes. This argument can be weakened by the consistent teaching mentality of our experienced surgeons toward residents, which leads to reliable and quick results (Zhu et al. 2021; Han et al. 2022). Furthermore, tumor surgeries are always led by a senior consultant.

The site of mandibular tumor infiltration and consequently the resection site appear to influence the postoperative quality of life. Resections including the symphysis are reported to achieve a lower postoperative quality of life (Warshavsky et al. 2019), which include tumor resection localizations of class S according to Urken’s classification. By contrast, B and R tumors appear to be associated with better quality of life (Warshavsky et al. 2019); however, our data revealed that the local recurrence rate is significantly higher (Table 6). This must be considered in the long-term evaluation of the quality of life with respect to recurring operations, which can lower the overall quality of life.

According to O’Brien et al., recurrences mostly emerged from soft tissues, especially in cases of incomplete resection with positive resection margins (O’Brien et al. 2003). In our cases, histological resection margins were mostly classified as R0 postoperatively; however, 13 cases were graded R1 and two R2, emphasizing the value of 3D soft tissue imaging and clinical examination after surgery in cases of mandibular tumor invasion.

## Conclusions

Lymph node recurrence and secondary lymph node metastasis rates were significantly higher in the group of marginal resections. No significant differences in local recurrences could be detected between marginal and segmental resection types, but initial tumor location had an impact on local and lymph node recurrence rates. Urken’s classification SB and S showed significantly fewer local and lymph node recurrences/metastases. As the occult metastasis rate remains high and recurrences predominantly emerge from soft tissues and rarely from the bones, close clinical examination and 3D imaging of soft tissues form an important combination to detect recurrences in this vulnerable patient population. Furthermore, we recommend a less invasive tumor ablation in the bone and a more radical resection in soft tissues.

## Data Availability

The data presented in this study are available on request from the corresponding author.
